# Induction of Arterial Inflammation by Immune Checkpoint Inhibitor Therapy in Lung Cancer Patients as Measured by 2-[^18^F]FDG Positron Emission Tomography/Computed Tomography Depends on Pre-Existing Vascular Inflammation

**DOI:** 10.3390/life14010146

**Published:** 2024-01-19

**Authors:** Raffaella Calabretta, Lucian Beer, Helmut Prosch, Daria Kifjak, Lucia Zisser, Patrick Binder, Stefan Grünert, Werner Langsteger, Xiang Li, Marcus Hacker

**Affiliations:** 1 Division of Nuclear Medicine, Department of Biomedical Imaging and Image-Guided Therapy, Medical University of Vienna, 1090 Vienna, Austriapatrick.binder@meduniwien.ac.at (P.B.);; 2 Division of Radiology, Department of Biomedical Imaging and Image-Guided Therapy, Medical University of Vienna, 1090 Vienna, Austria; 3 Department of Radiology, UMass Memorial Medical Center and University of Massachusetts Chan Medical School, Worcester, MA 01655, USA

**Keywords:** atherosclerosis, immune checkpoint inhibitors, PET, 2-[^18^F]FDG, lung cancer, cardio-oncology

## Abstract

Background: Immune checkpoint inhibitors (ICI) are one of the most effective therapies in oncology, albeit associated with various immune-related adverse events also affecting the cardiovascular system. Methods: We aimed to investigate the effect of ICI on arterial 2-[^18^F]FDG uptake by using 2-[^18^F]FDG PET/CT imaging pre/post treatment in 47 patients with lung cancer. Maximum 2-[^18^F]FDG standardized uptake values (SUVmax) and target-to-background ratios (TBRs) were calculated along six arterial segments. We classified the arterial PET lesions by pre-existing active inflammation (cut-off: TBRpre ≥ 1.6). 2-[^18^F]FDG metabolic activity pre/post treatment was also quantified in bone marrow, spleen, and liver. Circulating blood biomarkers were additionally collected at baseline and after immunotherapy. Results: ICI treatment resulted in significantly increased arterial inflammatory activity, detected by increased TBRs, in all arterial PET lesions analyzed. In particular, a significant elevation of arterial 2-[^18^F]FDG uptake was only recorded in PET lesions without pre-existing inflammation, in calcified as well as in non-calcified lesions. Furthermore, a significant increase in arterial 2-[^18^F]FDG metabolic activity after immunotherapy was solely observed in patients not previously treated with chemotherapy or radiotherapy as well as in those without CV risk factors. No significant changes were recorded in either 2-[^18^F]FDG uptake of bone marrow, spleen and liver after treatment, or the blood biomarkers. Conclusions: ICI induces vascular inflammation in lung cancer patients lacking pre-existing arterial inflammation.

## 1. Introduction

Cancer immunotherapy with immune checkpoint inhibitors (ICI), including monoclonal antibodies targeting cytotoxic T lymphocyte-associated antigen 4 (CTLA-4), programmed cell death protein 1 (PD-1) and its ligand (PD-L1), has revolutionized cancer treatment by enhancing immune responses against tumors [1]. Nevertheless, a major concern with their use is the non-specific activation of the immune system, which often precipitates immune-related adverse events (IRAEs), a spectrum of side effects distinct from traditional cancer treatments [2,3]. While the majority of these IRAEs manifest in the gastrointestinal tract, skin, or the endocrine system, there have also been notable reports of myocarditis, pericarditis, vasculitis, arrhythmias, and acute coronary syndromes [4,5,6].

Several recent studies have highlighted an elevated incidence of cardiovascular (CV) complications in cancer patients undergoing ICI therapy. Some of these complications can be attributed, at least in part, to the acceleration of atherosclerotic processes [7,8,9,10]. Atherosclerosis, an enduring inflammatory affliction targeting the arterial wall, is heavily influenced by the activities of monocytes and macrophages. Even brief exposure to inflammatory triggers typical of atherogenesis can induce a sustained inflammatory hyperactivation of innate immune cells, potentially exacerbating atherosclerosis [11]. Given that cardiovascular diseases (CVD) rank as the primary cause of global mortality, pinpointing individuals particularly susceptible to cardiac or CV toxicity subsequent to cancer immunotherapy is of paramount importance [12,13,14]. The concern is even more pronounced for lung cancer patients, as their smoking history may predispose them to accelerated atherosclerosis [15]. An established approach to probe and quantify atherosclerotic inflammation is positron emission tomography (PET) employing 2-[^18^F]fluoro-d-glucose (2-[^18^F]FDG), also in oncological patients [16,17]. Recently, we described elevated inflammatory activity in large arteries of two patient cohorts treated with ICI, suffering, respectively, from melanoma and lymphoma, using 2-[^18^F]FDG-PET imaging [18,19]. Yet, the precise molecular dynamics governing atherosclerotic inflammation after ICI treatment in cancer patients remain unknown. In our present research, we delve deeper into the ramifications of pre-existing vascular inflammatory load on the arterial inflammatory reaction following ICI treatment. We included a cohort of lung cancer patients, anticipating a higher inflammatory burden owing to smoking and other associated CVD risk factors [15]. Using 2-[^18^F]FDG signal intensity as a surrogate biomarker for inflammation, we investigated the arterial effects of immunotherapy in lesions with and without pre-existing elevated 2-[^18^F]FDG uptake. Furthermore, the cohort was subdivided based on their exposure to pro-inflammatory factors like chemotherapy (CHT), radiotherapy (RT), and concomitant CVD risk factors. Although there is still no standard method for the identification and evaluation of the organ inflammation after immunotherapy exposure on 2-[^18^F]FDG PET imaging [20,21], our study provides important insights into the risk of CV toxicity associated with cancer immunotherapy.

## 2. Materials and Methods

A cohort of 47 patients with histologically confirmed lung cancer, some of whom had been enrolled in a previous prospective protocol [22], underwent 2-[^18^F]FDG positron emission tomography–computed tomography (PET/CT) scans both prior to and during ICI treatment at the Division of Nuclear Medicine at the Medical University of Vienna. All 2-[_18_F]FDG PET/CT examinations were performed with the same scanning protocol using a 64-multi–detector-row hybrid PET/CT device (Biograph TruePoint 64; Siemens Healthineers, Erlangen, Germany), as previous described. This retrospective single-center study was conducted under the auspices of our institutional review board (Ethics Committee of the Medical University of Vienna—Approval no. 1367/2020) and conformed to the Declaration of Helsinki’s guidelines. The time interval between pre- and post-PET scans was 2.5 ± 1 month. Baseline patient characteristics and CVD risk factors (Table 1). 

Oncological data, including previous anti-cancer treatments such as CHT and RT, and available blood biomarkers were collected (Table 2). 

For PET image data analysis, 2-[^18^F]FDG maximum standardized uptake values (SUV_max_) were quantified along six arterial segments (aortic arch, ascending, descending and abdominal aorta, iliac arteries) by placing 1 cm^3^ cubic volumes of interest (VOIs). We analyzed the most diseased arterial lesions in PET scans before and after therapy, according to one of the validated methods previously described [16]. 2-[^18^F]FDG SUV_max_ values were corrected for 2-[^18^F]FDG blood-pool activity (SUV_bloodpool_), which was derived by positioning three cubic 1 cm^3^ VOIs within the lumen of the superior vena cava and the mean SUV was calculated. The target-to-background ratios (TBRs) were subsequently derived as previously reported, by correcting 2-[^18^F]FDG SUV_max_ values for SUV_bloodpool_ [16,23]. After that, considering a TBR threshold of >1.6 as significant for the active segment analysis [16], we classified the arterial PET lesions by pre-existing active inflammation (cut-off: TBRpre ≥ 1.6). On the CT images, we performed a lesional calcium density categorization and divided them into calcified (≥130 Hounsfield Units—HU) and non-calcified (<130 HU) [23]. 

Continuous variables were recorded as mean ± SD. Differences in mean uptake values of 2-[^18^F]FDG (SUV_max_ and TBR) pre- and post-therapy were retrospectively assessed using the paired Student’s *t*-test. Subsequently, the change in TBR values (ΔTBR = TBR_post_ − TBR_pre_) was calculated and compared using the ANOVA test. Two-sided *p*-values of <0.05 were considered significant. 

Additionally, 2-[^18^F]FDG uptake as SUVmean was measured before and after ICI therapy in the spleen and bone marrow as surrogate markers for systemic immune cell activation. Here, bone marrow SUVmean was quantified by placing manual regions of interest (ROIs) on each vertebra in axial projections of thoracic and lumbar spines. Splenic 2-[^18^F]FDG SUVmean was assessed by positioning three manual ROIs in coronal, axial, and longitudinal projections of the organ parenchyma [24]. Also, hepatic SUVmean was recorded, by placing three manual ROIs in coronal, axial, and longitudinal projections of the organ parenchyma. Although some IRAEs will not necessarily be associated with clinical symptoms, a few could potentially be identified on 2-[^18^F]FDG PET images by the inversion of the spleen-to-liver ratio (SLR—normally > 1), which was also calculated in all patients as a ratio between splenic SUVmean and hepatic SUVmean [24].

As further markers of systemic inflammation, high-sensitivity C-reactive protein (hsCRP) values before and after ICI treatments were collected. Additionally, absolute leukocyte counts (ALeC), absolute erythrocyte counts (AEC), and absolute platelet counts (APC), as well as absolute and relative neutrophil counts (ANC, RNC) and absolute and relative lymphocyte counts (ALC, RLC) pre- and post-therapy were recorded and analyzed. Since the neutrophil/lymphocyte ratio (NLR) could be an available inflammatory biomarker associated with atherosclerosis and might predict CV events, the absolute and relative NLRs before and after immunotherapy were derived [25]. 

## 3. Results

**Figure 1:**
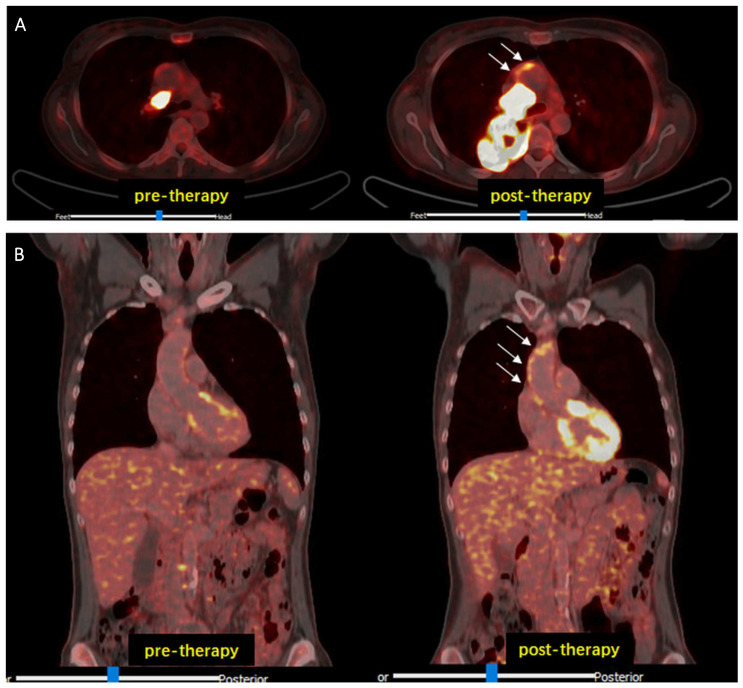
Representative 2-[18F]FDG PET/CT images. Increased arterial 2-[18F]FDG uptake (white arrows) after ICI therapy compared to baseline examination (pre-therapy) observed in transaxial (**A**) and in coronal (**B**) views. Abbreviations: CT, computed tomography; 2-[^18^F]FDG, 2-[^18^F]fluorodeoxyglucose; ICI, immune checkpoint inhibitors; PET, positron emission tomography.

Cancer immunotherapy with ICI resulted in a modest but significant increase in 2-[^18^F]FDG-uptake in all analyzed arterial PET lesions (n = 761; lesional TBR_pre_ = 1.73 ± 0.42 vs. lesional TBR_post_ = 1.90 ± 0.44; *p* < 0.001), interpreted as inflammatory activity (Figure 1). 

**Figure 2:**
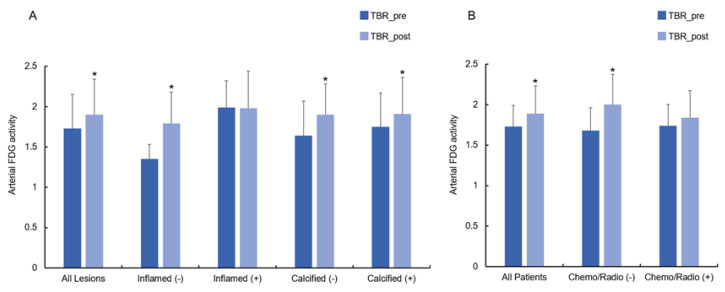
Change in TBRs in arterial lesions after immunotherapy with ICI. (**A**) Significant increase in TBRs was found in all arterial lesion analyzed. In particular, TBR values were significantly higher in the lesions without pre-existing arterial inflammation as well as in both non-calcified and calcified lesions. (**B**) Change in TBRs in patients after ICI immunotherapy. Significant increase in TBRs was registered in all patients. In particular, TBRs were significantly elevated after ICI in all patients without previous CHT or RT. Abbreviations: CHT, chemotherapy; ICI, immune checkpoint inhibitors; RT, radiotherapy; TBR, target-to-background ratio. * Significance of *p* < 0.05.

In per-patient analysis, there was also significantly higher arterial inflammation after treatment compared to baseline (n = 47; lesional TBR_pre_ = 1.73 ± 0.26 vs. lesional TBR_post_ = 1.89 ± 0.34; *p* < 0.001). After therapy, TBRs were significantly elevated in lesions without pre-existing arterial inflammation (n = 305; TBR_inf(−)_pre_ = 1.35 ± 0.18 vs. TBR_inf(−)_post_ = 1.79 ± 0.39; *p* < 0.001), while no further elevation was observed in PET lesions with pre-existing active inflammation (n = 456; TBR_inf(+)_pre_ = 1.99 ± 0.33 vs. TBR_inf(+)_post_ = 1.98 ± 0.46; *p* = 0.77) (Figure 2A). Regarding the lesional calcium density categorization, a significant increase in TBRs was noted in both calcified (n = 73; TBR_calc(+)_pre_ = 1.75 ± 0.42 vs. TBR_calc(+)_post_ = 1.91 ± 0.45; *p* < 0.001) and non-calcified lesions (TBR_calc(−)_pre_ = 1.64 ± 0.43 vs. TBR_calc(−)_post_ = 1.99 ± 0.38; *p* < 0.001) (Figure 2A). 

**Figure 3:**
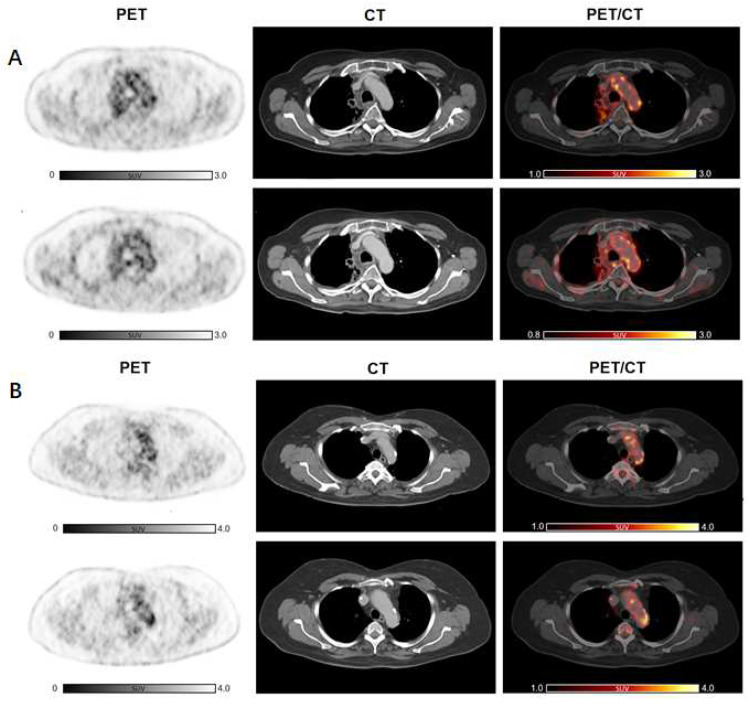
2-[^18^F]FDG arterial metabolic activity after ICI therapy compared to baseline in patients previously treated with other anti-cancer therapies. No significant increase in arterial 2-[^18^F]FDG uptake after immunotherapy can be detected in a subject already treated with RT (**A**) as well as in a patient who earlier underwent CHT and RT (**B**). Abbreviations: CHT, chemotherapy; 2-[^18^F]FDG, 2-[^18^F]fluoro-d-glucose; ICI, immune checkpoint inhibitors; RT, radiotherapy.

**Figure 4:**
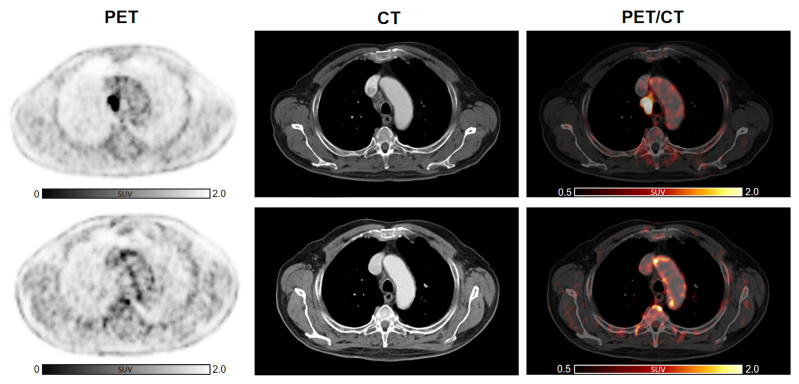
Increased 2-[^18^F]FDG arterial uptake after ICI therapy compared to baseline in a subject without any other previous anti-cancer treatment. Cancer immunotherapy with ICI resulted in a modest but significant increase in arterial 2-[^18^F]FDG in a patient not previously treated with other anti-cancer therapies, such as CHT or RT. Abbreviations: CHT, chemotherapy; 2-[^18^F]FDG, 2-[^18^F]fluoro-d-glucose; ICI, immune checkpoint inhibitors; RT, radiotherapy.

Subsequently, we analyzed the change in arterial inflammatory activity by dividing the patients into two groups, according to the presence of previous anti-cancer treatments, such as CHT or RT, and with presence/absence of CVD risk factors. Cancer immunotherapy with ICI resulted in a significant increase in inflammatory activity in patients without previous CHT (n = 19, TBR_CHT(−)_pre_ = 1.64 ± 0.26 vs. TBR_CHT(−)_post_ = 1.91 ± 0.36; *p* < 0.001). In patients treated previously with CHT, TBRs remained substantially unchanged (n = 28, TBR_CHT(+)_pre_ = 1.79 ± 0.25 vs. TBR_CHT(+)_post_ = 1.88 ± 0.34; *p* = 0.18) (Figure 2B). Furthermore, significantly elevated TBR values were recorded after therapy in the subjects without previous RT (n = 25, TBR_RT(−)_pre_ = 1.68 ± 0.25 vs. TBR_RT(-)_post_ = 1.93 ± 0.38; *p* < 0.001), while no significant changes were observed in patients with prior RT (n = 22, TBR_RT(+) _pre_ = 1.78 ± 0.26 vs. TBR_RT(+)_post_ = 1.84 ± 0.29; *p* = 0.34) (Figure 2B). Representative images are shown in Figure 3 as well as in Figure 4. 

TBRs increased significantly in patients without CVD risk factors (n = 29, TBR_RF(−)_pre_ = 1.72 ± 0.28 vs. TBR_RF(−)_post_ = 1.89 ± 0.34; *p* < 0.01), but remained unchanged in patients with CVD risk factors (n = 18, TBR_RF(+)_pre_ = 1.73 ± 0.24 vs. TBR_RF(+)_post_ = 1.90 ± 0.37; *p =* 0.12). 

**Figure 5:**
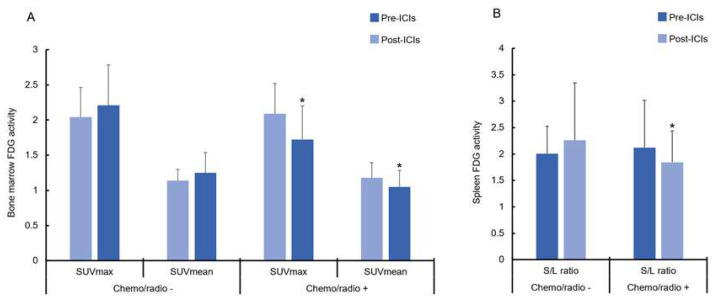
(**A**) Comparison between the change in 2-[^18^F]FDG metabolic activity in bone marrow before and after ICI therapy. A significant decrease in metabolic activity in bone marrow, interpreted as SUV_max_ and SUV_mean_, was recorded in all patients previously treated with CHT or RT. (**B**) SLR after ICI therapy. A significant decrease in metabolic activity in bone marrow, interpreted as SUVmax and SUVmean, was recorded in all patients previously treated with CHT or RT. Abbreviations: CHT, chemotherapy; 2-[^18^F]FDG, 2-[^18^F]fluoro-d-glucose; ICI, immune checkpoint inhibitors; RT, radiotherapy; SLR, spleen-to-liver ratio; SUV, standardized uptake value. * Significance of *p* < 0.05.

No significant changes were recorded in 2-[^18^F]FDG metabolic activity of the spleen (SUV_mean_spleen_pre_ = 1.77 ± 0.47 vs. SUV_mean_spleen_post_ = 1.78 ± 0.37; *p =* 0.92), bone marrow (SUV_mean_BM_pre_ = 1.17 ± 0.20 vs. SUV_mean_BM_post_ = 1.12 ± 0.27; *p =* 0.34), and liver (SUV_mean_liver_pre_ = 1.99 ± 0.33 vs. SUV_mean_liver_post_ = 2.02 ± 0.41; *p =* 0.61). Only the maximum 2-[^18^F]FDG-SUV values in the bone marrow were significantly lower after ICI compared to baseline (SUV_max_BM_pre_ = 2.07 ± 0.42 vs. SUV_max_BM_post_ = 1.89 ± 0.56; *p* < 0.09). When separating the patients into two groups (previous CHT/RT vs. no previous CHT/RT), we recorded that patients with previous anti-cancer treatments had significantly lower metabolic bone marrow activity after ICI therapy for both SUV_max_ (SUV_max_BM_CHT/RT(+)_pre_ = 2.09 ± 0.43 vs. SUV_max_BM_CHT/RT(+)_post_ = 1.73 ± 0.47; *p* < 0.006) and SUV_mean_ (SUV_mean_BM_CHT/RT(+)_pre_ = 1.18 ± 0.21 vs. SUV_mean_BM_CHT/RT(+)_post_ = 1.05 ± 0.24; *p =* 0.036) (Figure 5A). Conversely, the SUV_max_ (SUV_max_BM_CHT/RT(+)_pre_ = 2.09 ± 0.43 vs. SUV_max_BM_CHT/RT(−)_post_ = 2.04 ± 0.42; *p =* 0.40) and SUVmean (SUV_mean_BM_CHT/RT(−)_pre_ = 1.14 ± 0.16 vs. SUV_mean_BM_CHT/RT(−)_post_ = 1.24 ± 0.29; *p =* 0.27) in patients without prior CHT or RT were essentially unchanged (Figure 5A). 

SLRs after therapy were unchanged across the entire patient cohort (SLR_pre_ = 2.09 ± 0.80 vs. SLR_post_ = 1.97 ± 0.80; *p =* 0.33). However, significantly lower SLR values were measured in patients who underwent previous CHT or RT (SLR_CHT/RT(+)_pre_ = 2.12 ± 0.89 vs. SLR_CHT/RT(+)_post_ = 1.84 ± 0.59; *p =* 0.03), compared to patients without (SLR_CHT/RT(−)_pre_ = 2.01 ± 0.52 vs. SLR_CHT/RT(−)_post_ = 2.26 ± 1.09; *p =* 0.38) (Figure 5B). 

No significant alteration between before and after ICI was observed in any blood biomarker collected (Table 3).

## 4. Discussion

Atherosclerosis is intrinsically a chronic inflammatory pathophysiology characterized by the deposition of lipid-laden and immunological cells within the intimal layers of medium to large arteries. The underlying vascular inflammatory processes have an enormous impact on public health, and numerous metrics and biomarkers associated with atherosclerosis have been extensively studied in different patient cohorts and clinical settings [12,13,16]. However, circulating biomarkers currently fail to predict atherosclerotic progression. Furthermore, there is not currently a standard definition for the assessment of ICI immunotherapy-induced organ inflammation on 2-[^18^F]FDG PET imaging [20,21]. However, the imaging-based detection of arterial inflammation is thus an attractive approach to identify patients at high CVD risk [16,26,27,28,29]. Notably, the characterization of activated immune cells using PET imaging represents an interesting method for analyzing morphological and biological aspects of atherosclerotic plaques and identifying patients at higher risk for atherosclerotic CV events [16,26,27,28,29]. The recognized efficacy of 2-[^18^F]FDG PET in atherosclerosis diagnosis further underscores its importance [17,29]. 

In the present study, we retrospectively analyzed 2-[^18^F]FDG PET/CT image data before and after ICI treatment of 47 lung cancer patients, as an early readout of possible arterial inflammation after short-term therapy exposure. 2-[^18^F]FDG is a glucose analog that is taken up by cells with high metabolic activity, such as inflammatory cells, and its accumulation in the arterial wall is thought to reflect local increased inflammation [16]. All analyzed PET lesions displayed a significant increase in arterial 2-[^18^F]FDG metabolic activity after immunotherapy, interpreted as atherosclerotic inflammation, confirming our previous findings regarding melanoma and lymphoma patients, also treated with ICI [18,19]. In particular, we recorded higher TBR values after therapy in PET lesions without pre-existing arterial inflammation, while no further significant elevation was observed in lesions with pre-existing active inflammation. Moreover, we observed a significant increase in arterial TBR in patients not previously treated with CHT and/or RT and in those without CVD risk factors. Based on these findings, we speculate that “inflammation naive vessels” could be more sensitive to ICI treatment compared to the “already inflamed vessels”. The latter predominantly being found in patients who underwent previous anti-cancer treatments or presented with CVD risk factors. As mentioned before, after short-term exposure to atherogenic inflammatory stimuli, cells from the innate immune system can enter a state of elevated inflammatory activity, which may contribute to the development and progression of atherosclerosis [11]. Indeed, patients treated with CHT or RT as well as subjects presenting atherosclerotic CVD risk factors before starting of ICI treatment have higher lesional baseline TBRs and hence a pre-existing arterial inflammatory activity. Walker et al. had previously explained as inflammation is a well-known consequence of different traditional anticancer treatments and can enhance the antitumor immunity and promote unexpected side effects, which could lead to a chronic low-grade systemic inflammation [30]. We hypothesize that exactly the pre-existing inflammatory activity recorded in the arterial PET lesions could hamper an additional pro-inflammatory effect of immunotherapy on arterial walls resulting in a modest, but not significant, increase in 2-[^18^F]FDG arterial uptake [11,31,32]. Still, the treatment with CHT and RT may contribute to modifying the tumor microenvironment by decreasing immunosuppression and breaking the self-tolerance of the tumor, with subsequent enhancement of immunotherapy over the tumor self [33], as explained by Kershaw et al. [34]. Conversely, patients who did not undergo previous CHT or RT or were lacking CVD risk factors still possess a higher inflammatory reserve and show a higher inflammatory atherosclerotic activity after ICI therapy. The attention of clinicians should focus precisely on this last group of patients with newly active arterial inflammation after ICI, as their higher risk of developing atherosclerotic CV events may be overlooked. Indeed, macrophages resident in plaques can present different phenotypes, including subsets associated with plaque vulnerability. In accordance with prior findings [8,9], the amplified immune response subsequent to immunotherapy may result in local higher inflammation in atherosclerotic plaques, possibly leading to their destabilization and the occurrence of atherosclerotic or acute CV events [18,32,35]. Previous preclinical and clinical studies indeed described a correlation between ICI and atherosclerosis, suggesting activated T-cells to produce large amounts of pro-atherogenic cytokines that might contribute to both growth and destabilization of atherosclerotic plaques [8]. Furthermore, Drobni et al. recently demonstrated that ICI therapy was associated with an increased incidence of CV events compared to the control group in a large cohort, potentially mediated by an accelerated progression of atherosclerosis, which is concordant with our findings [9]. 

Considering the lesion calcium density categorization, we interestingly observed a significant increase in TBRs in both calcified and non-calcified lesions. Vascular microcalcifications have already been studied as a possible vulnerability and inflammation factor for atherosclerotic plaque [36]. Indeed, Wen et al. explained as the assessment of microcalcification in vascular plaques by using PET/CT with [^18^F]sodium fluoride ([^18^F]NaF) is a valid tool for detecting high-risk coronary plaque and for improving the risk stratification of these patients [36].

Regarding lymphoid organs, no significant alterations in 2-[^18^F]FDG metabolic activity post-ICI PET scans were recorded. Nevertheless, still, a perceptible decline in 2-[^18^F]FDG metabolic vigor was noticed in bone marrow, notably in patients with a history of CHT or RT. This trend may underscore a localized immunomodulatory sway on bone marrow progenitors of mature innate immune cells [11]. 

Despite hsCRP levels being marginally elevated post-treatment, the overall significance eluded us, aligning with observations by Denegri et al. [37] and of Soeki et al. [38]. However, given the multifaceted influencers on hsCRP, including smoking, tumor milieu, and chronic stress, its role as a specific active atherosclerosis indicator remains contentious [37,38,39] and it might not be considered as a specific marker of active atherosclerotic inflammation. Hence, the value of hsCRP for monitoring of atherosclerosis progression remains limited [37,38,39]. Furthermore, also the NLR, recently appointed as an autonomous herald of atherosclerotic CVD risk, did not display marked fluctuations in our cohort. 

Therefore, despite the lack of a standard definition for the assessment of organ inflammation induced by ICI immunotherapy on PET imaging with 2-[^18^F]FDG [20,21], our results suggested that this imaging modality, often performed in clinical practice for oncological diagnostic purposes, could be a valid tool to detect early the onset or the progression of atherosclerosis in cancer patients.

In summary, our findings allow us to hypothesize that cancer immunotherapy with ICI could markedly impinge upon the major arteries, particularly in patients devoid of a prior vascular inflammatory activation potentially induced by chronic inflammatory cues. Although the main limitations of this study are the small patient population and the lack of clinical follow-up and event data, the insights gleaned might be instrumental in early patient stratification, spotlighting those at heightened risk of CV toxicity post-ICI immunotherapy. 

## 5. Conclusions

Our investigation illuminates a pivotal finding: patients with minimal pre-exposure to inflammatory stimuli prior to the initiation of ICI therapy exhibit an augmented immune response, paralleled by a pronounced increment in arterial inflammation post-treatment. This is potentially attributable to the preservation of immune reserve in these individuals. Conversely, patients with pre-existing arterial inflammation do not manifest significant progression in atherosclerotic inflammatory activity post-ICI therapy. These insights underscore the differential immunovascular responses contingent upon the pre-therapeutic inflammatory status of the cancer patients undergoing ICI regimens.

However, further studies on cancer patients receiving ICI through the use of new alternative targets and tracers for imaging inflammation in CVD as well as of a novel PET/CT whole-body scanner are still needed to increase our knowledge in this field of research.

## Figures and Tables

**Table 1:** Baseline patient characteristics.

Gender (males/females)	28/19
Age (years, mean ± SD)	61 ± 9
BMI (kg/m^2^, mean ± SD)	26 ± 3.7
Smoking, N (%)	31 (70)
Hypertension, N (%)	16 (34)
Dyslipidemia, N (%)	4 (8.5)
Diabetes, N (%)	4 (8.5)
COPD, N (%)	16 (34)
Prior myocardial infarction, N (%)	4 (8.5)
Prior TIA/Stroke, N (%)	4 (8.5)
PAD, N (%)	8 (17)

Abbreviations: BMI, body mass index; COPD, chronic obstructive pulmonary disease; PAD, peripheral artery disease; TIA, transient ischemic attack.

**Table 2:** Oncological features of patient cohort.

Histology (N; %)	Lung adenocarcinoma (33; 70.2) Lung squamous cells carcinoma (14; 29.8)
Tumor stadium (N; %)	I (0; 0) II (0; 0) IIIA/IIIB (6; 12.8) IV (41; 87.2)
ICI Therapy (N; %)	47; 100 PD-1 Inhibitors (26; 55.3) Nivolumab (7; 14.9) Pembrolizumab (19; 40.2) PD-L1 Inhibitors (12; 25.5) Atezolizumab (8; 17) Durvalumab (4; 8.5) Combination of ICI + CHT (9; 19.1)
Previous ICI Therapy, N (%)	1 (2.1)
CHT before ICI therapy, N (%)	28 (59.6)
RT during ICI therapy, N (%)	1 (2.1)
RT before ICI therapy, N (%)	22 (46.8)
Previous surgery, N (%)	16 (34)
PD-1 Expression > 50%, N (%)	25 (53.2)
PD-1 Expression ≤ 50%, N (%)	22 (46.8)

Abbreviations: CHT, chemotherapy; ICI, immune checkpoint inhibitor; PD-1, programmed cell death 1; PD-L1, programmed cell death 1-ligand; RT, radiotherapy.

**Table 3:** Blood biomarkers.

	Pre	Post	*p* Value
hsCRP	22.04 ± 27.19	22.61 ± 6.75	0.887
ALeC	7.97 ± 3.22	7.94 ± 3.03	0.957
AEC	4.36 ± 0.56	4.36 ± 0.53	0.924
APC	285.20 ± 130.01	295.37 ± 111.93	0.550
ANC	5.49 ± 2.59	5.33 ± 2.72	0.700
RNC	66.37 ± 11.28	64.98 ± 11.24	0.338
ALC	1.66 ± 0.86	1.62 ± 0.78	0.676
RLC	21.32 ± 9.39	22.00+10.43	0.550
NLR absolute	4.32 ± 3.60	4.27 ± 3.47	0.921
NLR relative	4.32 ± 3.60	4.26 ± 3.46	0.911

Abbreviations: AEC, absolute erythrocytes count; ALC, absolute lymphocytes count; ALeC, absolute leukocytes count; ANC, absolute neutrophils count; APC, absolute platelets count; hsCRP, high sensitivity C-reactive protein; ICI, immune checkpoint inhibitor; NLR, neutrophil/lymphocyte ratio; RLC, relative lymphocyte counts; RNC, relative neutrophil counts.

## Data Availability

Data are contained within the article.
